# A novel classification, management and long-term outcomes of coronary artery involvement in acute aortic dissection

**DOI:** 10.1186/s12872-023-03301-z

**Published:** 2023-06-21

**Authors:** Yi Chang, Hongwei Guo, Cuntao Yu, Xiaogang Sun, Kan Yang, Xiangyang Qian

**Affiliations:** 1 Department of Vascular Surgery, Fuwai Hospital, Chinese Academy of Medical Science & Peking Union Medical College, National Center for Cardiovascular Diseases, No.167 North Lishi Road, Xicheng District, Beijing, China; 2 Department of Cardiovascular Surgery, Nanyang central hospital, No. 312 Gongnong Road, Wancheng District, Nanyang, Henan Province China

**Keywords:** Aortic dissection, Coronary artery

## Abstract

**Background:**

To introduce a new and simple classification and management of coronary artery involvement in aortic dissection and report results.

**Methods:**

Coronary artery involvement was classified into two types according to the integrity of coronary intima: simple lesion (type S) and complex lesion (type C). Complex lesions were treated by CABG and simple lesions were treated by ostial repair or reimplantation. Data were collected and analyzed retrospectively.

**Results:**

From January 2010 to December 2019, 267 consecutive patients were enrolled in the study, and among them complex lesions occurred in 27 patients (11.1%) and simple lesions was found in 240 patients(89.9%). Eleven untreated vessels with simple lesion were found to be involved again in the same operation and treated by CABG. The two type groups had comparable operative mortality (type S vs. type C, 9.6% vs. 18.5%, P = 0.28). 221 patients received follow-up with a median duration of 52 months. The overall survival rates at 1, 5, and 10 years postoperatively were 88.9%, 85.7%, and 79.8% in type S group and 79.2%, 79.2%, and 79.2% in type C group, respectively (P = 0.47). For the patients who received CABG and survived at discharge, radiographic follow-up with a median duration of 28 (IQR 7-55.5) months showed the freedom from occlusion of vein graft at 1, 5, and 10 years postoperatively were 87.5%, 70.0%, 28.0%.

**Conclusions:**

According to the new classification, two types of lesions could be treated by corresponding methods with satisfactory early and long-term results. Unrepaired coronary artery was at a risk of re-involvement. Vein graft onto arteries without atherosclerosis still had a high occlusion rate.

**Supplementary Information:**

The online version contains supplementary material available at 10.1186/s12872-023-03301-z.

## Introduction

Acute type A aortic dissection (ATAAD) is a life-threatening cardiovascular disease, especially when multiple branches are involved and consequent organ malperfusion occurs. It is reported that coronary artery involvement secondary to ATAAD is approximately 7- 20.7% [1, 2]. Neri’s classification [3] was used to evaluate severity of coronary artery involvement in the literatures but was not practical to guide management. How to deal with the involved coronary artery is crucial, unfortunately there was neither consensus on the optimal approach nor large sample study comparing different management. For affected coronary arteries, Neri et al. [3] proposed direct coronary artery repair superior to coronary artery bypass grafting (CABG), whereas Kawahito [4] recommended CABG in all types. We were sometimes confused about how to deal with involved coronary artery and adopted some therapeutic methods. So, based on summarization of previous cases, in this study we aim to: first, introduce a new and simple classification of coronary artery involvement; second, demonstrate the perioperative and long-term results.

## Patients and methods

### Ethics statement

This study had been approved by institutional review board (IRB) of Fuwai hospital, Peking union medical college and Chinese academy of medical sciences. All the consents of patients had been obtained. The IRB approval number is 2020 − 1402, the date is November 24, 2020.

### Study population

The database of aortic dissection of our institute was reviewed. Among the 1711 consecutive patients who were surgically treated for ATAAD from 2010 January to 2019 December in our center, patients with coronary artery involvement were enrolled in the study. The data of demographics, clinical features, imaging materials, surgical characteristics, postoperative outcomes and follow-up results were collected.

### Classification of coronary artery involvement

**Fig. 1:**
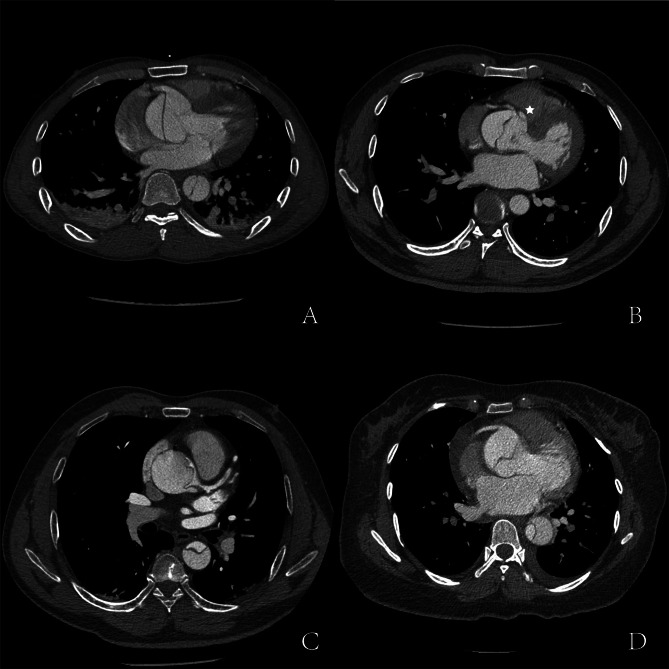
Manifestations of coronary artery involvement on computed tomography angiography **A**, type S1, dissection involving half of the ostium (right coronary) **B**, type S1, dissection involving a circle of the ostium; asterisk, false lumen under the ostium(right coronary) **C**, type S2, dissection extending to coronary trunk with thrombosis in false lumen(left coronary) **D**, type C2, coronary trunk originated from false lumen(right coronary)

**Fig. 2:**
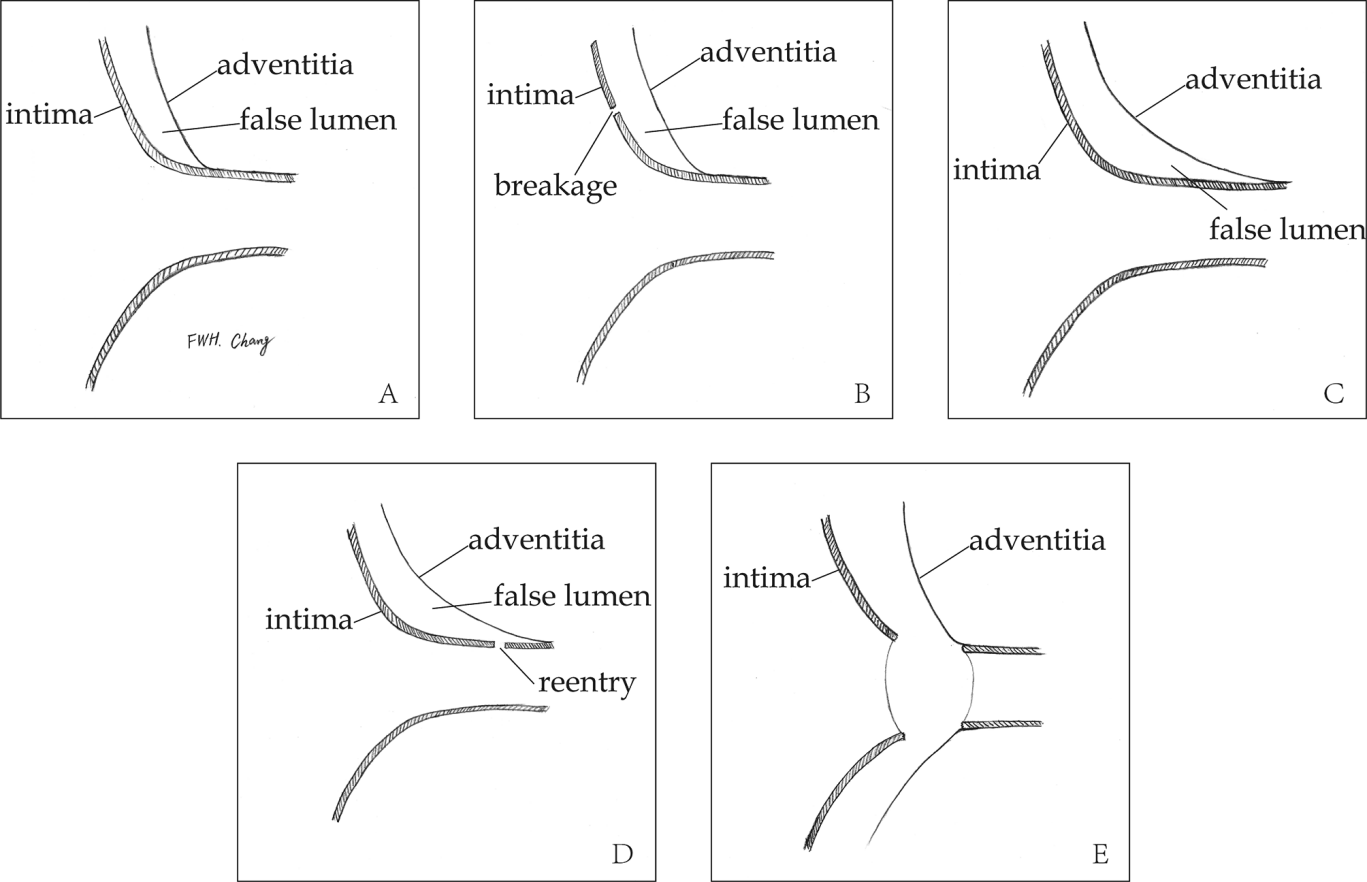
Classification and subgroups of coronary artery involvement **A**, type S1, dissection around ostium with intact intima around ostium **B**, type S2, dissection extending to coronary trunk with intact intima **C**, type C1, dissection extending to coronary trunk with distal reentries **D**, type C2, complete detachment of coronary trunk S = simple, C = complex

This classification was based on retrospective summarization of our cases. It was not a guideline of our practice in the past. Based on radiographic image and visual inspection in operation, coronary artery involvement could be classified into two types according to the integrity of coronary intima: simple lesion (type S) and complex lesion (type C). If intima was intact, no matter whether coronary trunk was dissected, it was denoted as type S. If there was distal reentry/tear or intimal detachment (partial or complete) of coronary trunk, it was denoted as type C. Complex lesion could be recognized clearly on computed tomography angiography (CTA) as coronary trunk originated from false lumen. The manifestations of coronary artery involvement on CTA were shown in Fig. 1. In some cases, distal reentry on coronary trunk was difficult to be found on CTA. When coronary trunk was involved on CTA as showed in Fig. 1-C, careful visual inspection was needed during operation to confirm whether there was a distal reentry. An indirect sign might be useful to identify distal reentry: when cardioplegia was infused via coronary ostium but returned to false lumen, there was likely to be a tear on distal trunk. We showed the two types and subgroups in Fig. 2.

### Institute-specific definition

Myocardial ischemia was defined as ST segment depression or elevation on electrocardiogram and elevated Troponin I. Hemodynamic instability was defined as persistent hypotension (systolic blood pressure < 90 mmHg) preoperatively for any reasons. Coronary artery disease (CAD) was diagnosed according to history and CTA. Operative mortality was defined as any death, regardless of cause, occurring within 30 days after surgery in or out of the hospital, and after 30 days during the same hospitalization subsequent to the operation.

### Surgical techniques

Method of cardioplegia infusion depended on the type of coronary artery involvement. Direct antegrade perfusion via ostium could be used for simple lesion. Immediate CABG and perfusion by vein graft was necessary for complex lesion. Retrograde perfusion was not used in our center during study period.

An aortic root replacement was generally applied in patients with a dilated aortic root (diameter > 45 mm), initial tear located in aortic root, or connective tissue disease. When aortic root could be reserved, kinds of approaches, as previous study reported [5–9], were used at surgeon’s discretion.

**Fig. 3:**
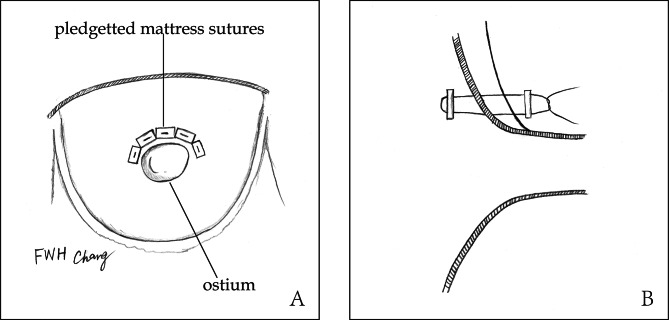
Ostial repair with intermittent pledgetted mattress stitches **A**, anterior view; **B**, lateral view

**Fig. 4:**
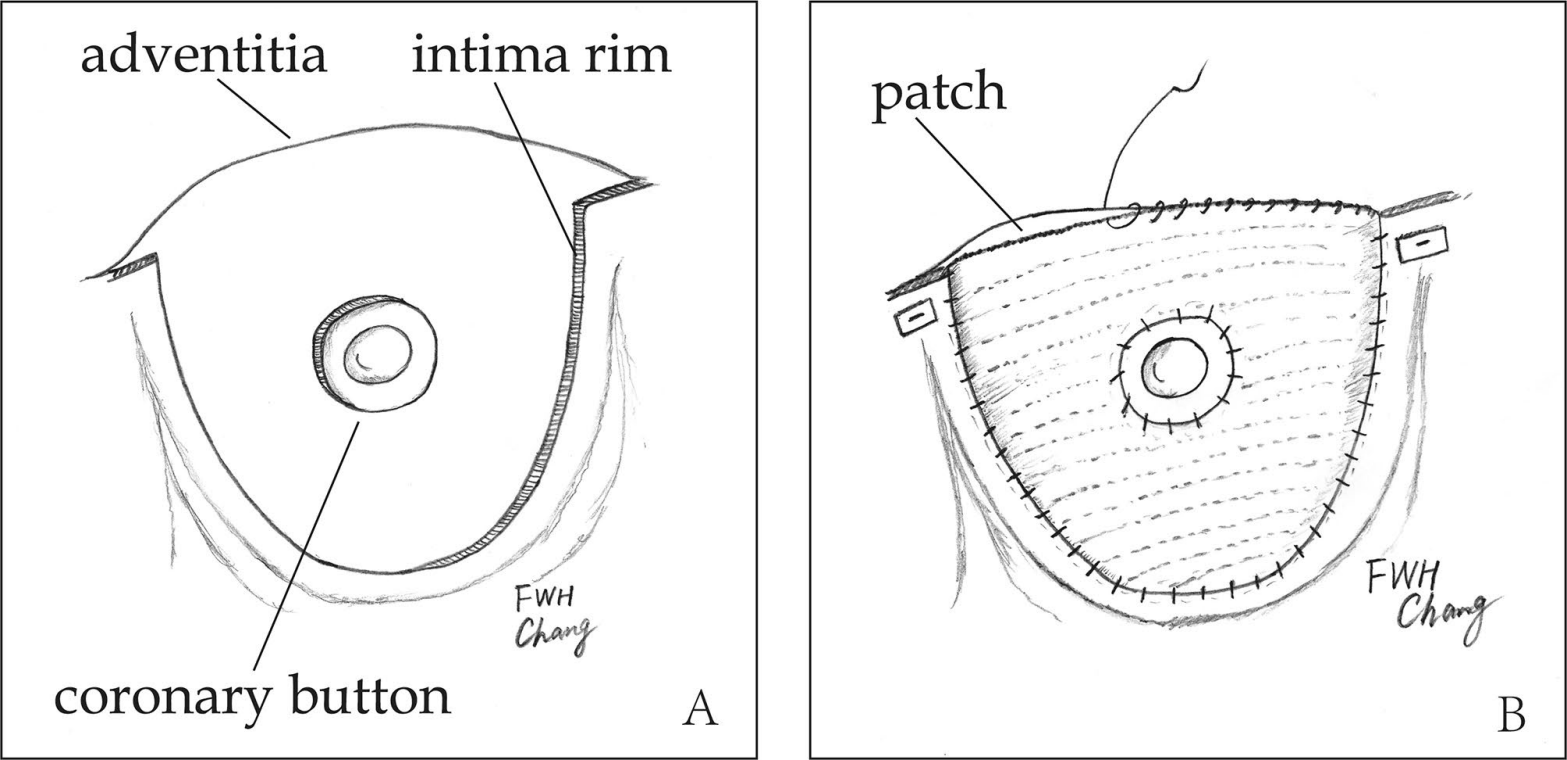
Single sinus replacement **A**, intima was resected with a rim of 5 mm wide and coronary ostium intima was trimmed into a button; **B**, a patch was sewn to the intima rim and coronary ostium was reimplanted

In cases of type S, management depended on surgeons’ experience. Ostial repair was performed as intermittent pledgetted mattress stitches were placed around the orifice from inside to outside of aorta (Fig. 3). Ostial reimplantation was used when root or single sinus was replaced (Fig. 4). In cases of type C, we performed CABG as a standard manner while the ostium was routinely oversewn. When coronary arteries were found to be involved again by recurrent dissection at aortic root in the same operation, CABG was performed. The main manifestations of re-involvement of coronary artery included: (1) hemodynamic instability: malignant arrythmia or failing to wean from cardiopulmonary bypass; (2) ECG change; (3) newly emerged abnormal wall motion observed by transesophageal echocardiography (TEE); (4) coronary ostium compressed by residual dissection detected by TEE. The patients with CAD were treated by CABG when there was evidence for significant coronary stenosis (>50%) on CTA. Considering the longer time of internal mammary artery harvesting, great saphenous vein graft was used routinely. Vein graft was anastomosed to prosthetic vessel proximally.

Total arch replacement (TAR) combined with frozen elephant trunk (FET) was applied in our institution to repair dissected arch and the indications were described by Sun et al. [10]. Distal first technique was routinely used. When the dissection was limited to ascending aorta or proximal arch, isolated ascending aorta replacement (AAR) or AAR with partial-arch replacement (PAR) was implemented. Arch replacements (either partial or total) were performed under deep (18 °C, early stage) or moderate (25 °C, later stage) hypothermia circulatory arrest, with unilateral antegrade selective cerebral perfusion (SCP).

### Follow up

Data was obtained from each patient’s medical chart during regular visits to the outpatient clinic or by telephone contact. The survivors received follow-up using CTA to evaluate the morphology of aorta and patency of vein graft. Survival and reintervention were investigated.

### Statistical analysis

Data were presented as mean and standard deviation for continuous data conforming to normal distribution and as number (%) for categorical data. Continuous variable that didn’t conform to a normal distribution were demonstrated as median and interquartile range (IQR). Mean of two continuous normally distributed variables were compared by independent samples student T test and χ2 test or the Fisher exact probability test (if necessary) was used to compare categorical variables. Kaplan-Meier method was used for survival analysis and log-rank test was used to compare the difference on survival rate and freedom from events. A value of P < 0.05 was considered significant. The data were analyzed by Stata (version 15.0, Stata Corp LP, College Station, TX, USA).

## Results

### Demographic and preoperative characteristics

Among the enrolled 267 patients, 202 (75.7%) patients were male, and the average age was 47.4 ± 9.8 years. Seven (2.6%) patients suffered preoperative myocardial ischemia and 21 (7.9%) patients had CAD. Aortic regurgitation >moderate occurred in 45 (16.9%) patients and hemodynamic instability was identified in 16 (6.0%) patients. Simple lesion was found in 240 (89.9%) patients, and complex lesion occurred in 27 (11.1%) patients, respectively. Single right coronary artery (RCA) was involved more frequently in 170 (63.7%) patients, on the other hand, 9 (3.4%) patients only had left coronary artery (LCA)involved. 88 (32.9%) patients had both coronary arteries involved. The other comorbidities were demonstrated in Table 1.

**Table 1:** Baseline information of the whole cohort

Variable	All N = 267	Type S N = 240	Type C N = 27	P value
Age (year, $\bar X$±SD)	47.4 ± 9.8	47.4 ± 9.9	48.0 ± 9.1	0.74
Gender (Male, n, %)	202(75.7)	187(77.9)	15(55.6)	0.01
Body weight (Kg, $\bar X$±SD)	77.2 ± 16.0	77.51 ± 16.5	74.59 ± 10.4	0.37
Time to operating room (hour, $\bar X$ ±SD)	21.4 ± 12.6	21.2 ± 12.0	18.1 ± 9.7	0.08
Hypertension (n, %)	160(59.9)	143(59.6)	17(63.0)	0.73
CAD (n, %)	21(7.9)	19(7.9)	2(7.4)	1.00
Diabetes mellitus (n, %)	3(1.1)	3(1.3)	0(0.00)	1.00
Prior stroke (n, %)	11(4.1)	9(3.8)	2(7.4)	0.31
Marfan syndrome (n, %)	24(9.0)	20(8.3)	4(14.8)	0.26
LVEF (%, $\bar X$ ±SD)	51.7 ± 6.1	51.8 ± 6.0	50.2 ± 6.3	0.18
AR (≥moderate) (n, %)	45 (16.9)	40(16.7)	5(18.5)	0.79
Any aortic stenosis (n, %)	5(1.9)	5(2.1)	0(0.00)	1.00
Myocardial ischemia (n, %)	7(2.6)	5(2.1)	2(7.4)	0.15
LCA involvement only	9(3.4)	8	1	-
RCA involvement only	170(63.7)	151	19	-
LCA + RCA involvement	88(32.9)	81	7*	-
Hemodynamic instability (n, %)	16(6.0)	13(5.4)	3(11.1)	0.21
Tamponade (n, %)	9(3.4)	8(3.3)	1(3.7)	1.00
visceral malperfusion (n, %)	35(13.1)	34(14.2)	1(3.7)	0.22

CAD, coronary artery disease; LVEF, left ventricle ejection fraction; AR, aortic regurgitation; LCA, left coronary artery; RCA, right coronary artery

*For these 7 patients, one coronary artery was type C, and the other one was type S

### Operative details and outcome characteristics

In the whole cohort, 73(27.4%) patients had aortic root replacement (71 Bentall procedure and 2 David procedure) and 4 (1.5%) patients received single sinus replacement. 261(97.8%) patients had TAR + FET and 6(2.2%) patients had isolated AAR or AAR + PAR. CABG was performed due to CAD in 13 patients who were all in type S group. The mean cardiopulmonary bypass (CPB) duration and cross-clamp duration in type C group was significantly longer than that in type S group (type C vs. type S, 288.3 ± 132.7 min vs. 220.7 ± 80.2 min, P<0.001 and 141.7 ± 33.3 min vs. 120.8 ± 35.2 min, P = 0.004, respectively). Twenty-eight patients (10.5%) died within 30 days after operation. Operative mortality was comparable between the two groups (type S vs. type C, 9.6% vs. 18.5%, P = 0.28). Two patients in type C group, while 9 patients in Type S group died of cardiogenic event. Four of seven patients (57.1%.) who had myocardial ischemia before operation died of circulatory failure (3 patients) or acidosis caused by visceral malperfusion (1 patients). Two patients received extracorporeal membrane oxygenation (ECMO) support but failed to be rescued ultimately. Incidence of postoperative myocardial infarction was comparable across the two groups (type S vs. type C, 1.3% vs. 3.7%, P = 0.35). Perioperative outcomes were showed in Table 2.

**Table 2:** Operative and outcome characteristics of the whole cohort

Variable	All N = 267	Type S N = 240	Type C N = 27	P value
Proximal repair (n, %)				0.442
Supracoronary aortic replacement	190(71.2)	167(69.6)	23(85.2)	
David procedure	2(0.8)	2(0.8)	0(0)	
Bentall procedure	71(26.6)	67(27.9)	4(14.8)	
Single sinus replacement	4(1.50)	4(1.67)	0(0)	
Distal repair (n, %)				0.37
AAR/AAR + PAR	6(2.2)	5(2.1)	1(3.7)	
TAR + FET	261(97.8)	235(97.9)	26(96.3)	
Concomitant CABG due to CAD	13(4.9)	13(5.4)	0(0)	NA
CPB duration (min, $\bar X$±SD)	227.5 ± 89.0	220.7 ± 80.2	288.3 ± 132.7	<0.001
Cross-clamp duration (min, $\bar X$±SD)	122.9 ± 35.5	120.8 ± 35.2	141.7 ± 33.3	0.004
SCP duration (min, $\bar X$±SD)	19.2 ± 6.9	19.0 ± 7.1	20.8 ± 3.8	0.18
MV duration (hour, $\bar X$±SD)	65.5 ± 108.7	59.2 ± 82.9	71.5 ± 232.3	0.75
ICU stay (day, $\bar X$±SD)	6.5 ± 12.1	6.4 ± 12.3	7.6 ± 10.2	0.63
Operative mortality (n, %)	28(10.5)	23(9.6)	5(18.5)	0.15
Heart-related death (n, %)	11(4.1)	9(3.8)	2(7.4)	0.31
ECMO (n, %)	2(0.8)	0(0.0)	2(7.4)	0.01
IABP (n, %)	2(0.8)	1(0.4)	1(3.7)	0.19
PMI (n, %)	4(1.5)	3(1.3)	1(3.7)	0.35
Stroke (n, %)	23(8.6)	20(8.3)	3(11.1)	0.71
Paraplegia (n, %)	14(5.2)	13(5.4)	1(3.7)	1.00
CRRT (n, %)	45(16.9)	38(15.8)	7(25.9)	0.18
Reoperation for bleeding (n, %)	19(7.1)	15(6.3)	4(14.8)	0.11
CABG as remedy approach (n, %)	11(4.1)	11(4.6)	0(0)	NA
Tracheotomy (n, %)	13(4.9)	11(4.6)	2(7.4)	0.63

AAR, ascending aorta replacement; PAR, partial arch replacement; TAR, total arch replacement; FET, frozen elephant trunk; CAD, coronary artery disease; CABG, coronary artery bypass grafting; SCP, selective cerebral perfusion; MV, mechanical ventilation; ICU, intensive care unit; ECMO, extracorporeal membrane oxygenation; IABP, intra-aortic balloon pump implantation; PMI, postoperative myocardial infarction; CRRT, continuous renal replacement therapy

P value is for comparison between type S group and type C group

### Therapeutic strategy for each involved coronary artery

Because 88 patients had both coronary arteries involved and the type and management of the two vessels were not always alike, in order to demonstrate more intuitive understanding of classification and corresponding management of each involved vessel, we described the characteristics in Table 3. In total, 355 coronary arteries (LCA 97 and RCA 258) were involved and among them 328 were type S. For type C, CABG and saphenous vein graft perfusion was applied for all the cases. For type S, antegrade perfusion via ostium was successfully achieved but management was diversified: one hundred and fifty-seven involved coronary arteries didn’t accept any management (LCA 41 and RCA 116); ostial repair and reimplantation were applied for 33 and 118 coronary arteries, respectively; oversewing the ostium combined with CABG was performed in 20 vessels without simultaneous atherosclerotic stenosis. Prophylactic CABG was implemented for 18 cases without atherosclerotic stenosis. 11 untreated coronary arteries of type S (in 11 patients) were found to be involved again and CABG was performed as a remedy approach for these patients and they successfully weaned from cardiopulmonary bypass (detailed information was showed in Additional File Table I). There was no coronary artery-related adverse event in other management groups. The management for different types was listed in Table 3.

**Table 3:** Characteristics of involved coronary artery (each coronary artery serves as a statistical unit)

Variables	Simple N = 328	Complex N = 27
Left coronary artery (n, %)	96 (29.3)	1 (3.7)
Right coronary artery (n, %)	232 (70.7)	26 (96.3 )
Myocardial ischemia ascribed to coronary artery involvement (n, %)	5 (1.5)	0 (0)
Atherosclerotic stenosis of involved coronary artery (n, %)	14 (4.3)	0 (0)
Cardioplegia	Antegrade perfusion	SVG perfusion
Management (n, %)		
No repair	157 (47.9)	0 (0)
Ostial repair	33 (10.1)	0 (0)
Ostial reimplantation	118 (36.0)	0 (0)
Oversew the ostium and CABG	20 (6.1)	27 (100)
Prophylactic CABG (n, %)	18 (5.5)	0 (0)
Recurrent coronary artery involvement (n, %)	11 (3.4)	0 (0)

CABG, coronary artery bypass grafting

### Follow-up results

**Fig. 5:**
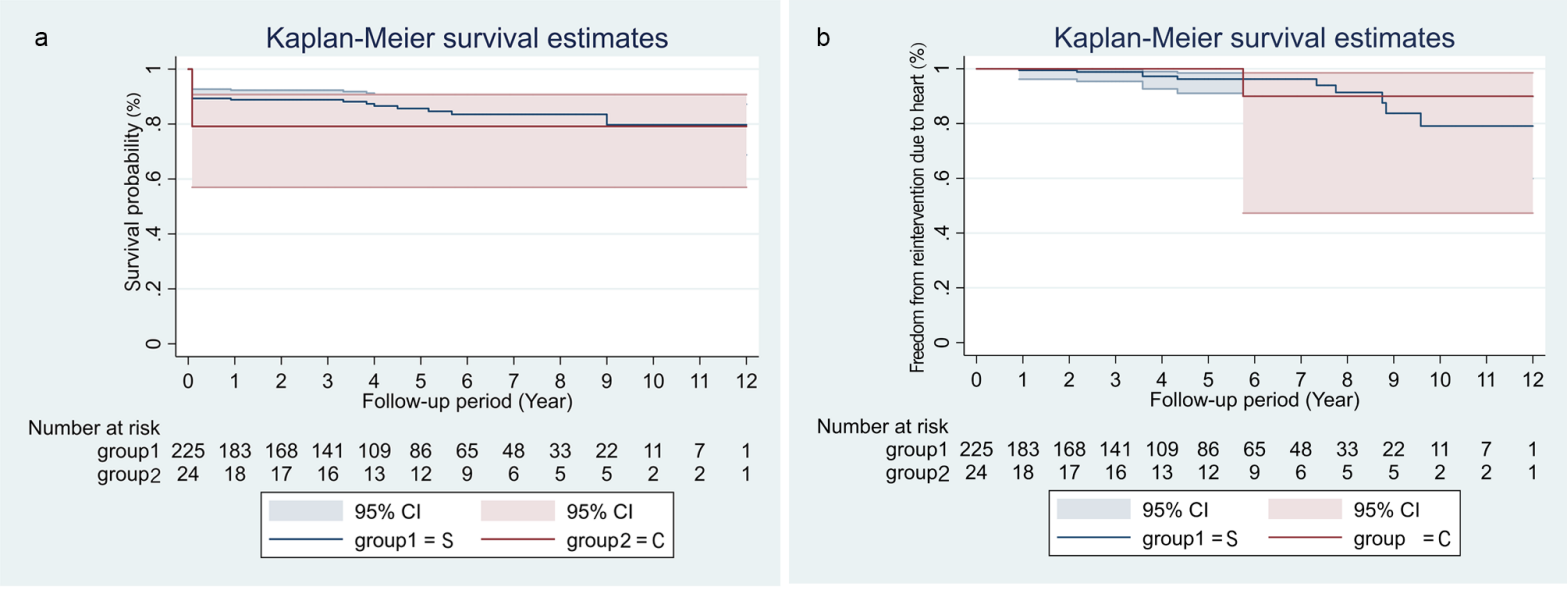
Survival analysis of two type groups Overall survival rate Freedom from reintervention due to heart S, simple type; C, complex type

**Fig. 6:**
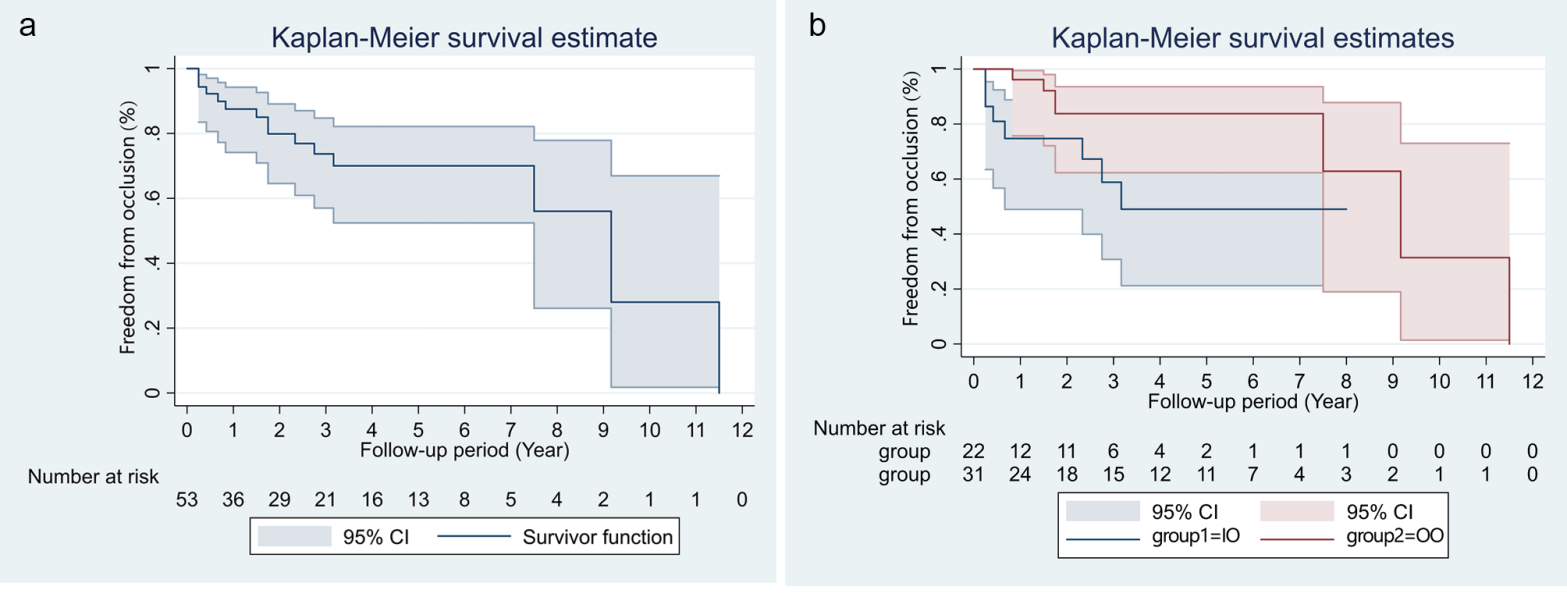
Freedom from occlusion Freedom from occlusion of vein graft in 53 patients Freedom from occlusion of vein graft by two groups, IO = intact ostium, OO = oversewn ostium

The median follow-up duration was 52 months (IQR 32–83). Twenty-eight patients died within 30 days in hospital and they were included in survival analysis. Eighteen patients were completely lost without any data and they were excluded from survival analysis. Two hundred and twenty-one patients accepted follow-up. During follow-up, all-cause death occurred in nine patients (all in type S group), including 3 cardiac deaths. Three patients died of unknown causes. The overall survival rates at 1, 5, and 10 years postoperatively were 88.9%, 85.7%, and 79.8% in type S group and 79.2%, 79.2%, and 79.2% in type C group, respectively. No significant differences in overall survival rates were found between the 2 groups (P = 0.47, Fig. 5A). Reintervention due to heart occurred in 11 patients (type S vs. type C, 10 vs. 1), including 3 percutaneous coronary interventions (PCI) for newly emerging CAD, 4 aortic root reoperations for residual dissection aneurysm, 2 aortic valve replacements, 1 mitral valve replacement, and 1 radiofrequency ablation for atrial flutter. The freedom from reintervention due to heart at 1, 5, and 10 years postoperatively were 99.5%, 96.2%, 79.1% in type S group, and 100%, 100%, 90.0% in type C group, respectively (P = 0.77, Fig. 5B). Of 74 patients received CABG due to coronary involvement, 58 patients survived at discharge and 53 patients received CTA follow-up. The median duration of radiographic follow-up was 28 (IQR 7-55.5) months. The overall freedom from occlusion of vein graft at 1, 5, and 10 years postoperatively were 87.5%, 70.0%, 28.0%, respectively (Fig. 6A). We categorized these 53 patients into two groups depending on whether the ostium was closed (IO = intact ostium, OO = oversewn ostium). The freedom from occlusion of vein graft was lower in IO group (IO vs. OO, 74.7% vs. 96.2% at 1 year, 49.1% vs. 83.8% at 5 years, P = 0.034, Fig. 6B). Thirteen patients received a radiographic follow-up longer than 60 months and graft occlusion occurred in 3 patients.

## Discussion

Coronary artery involvement is a troublesome condition that might result in myocardial ischemia before aortic repair operation. It is reported that incidence of coronary artery involvement is about 7–20.7% [1, 2, 11, 12], similarly in our database of ATAAD, the incidence was 15.6%, comparable to the literature. Myocardial ischemia caused by coronary artery involvement is a critical condition and develops in 5.7–11.3% of patients with ATAAD [11] and it may cause postoperative low cardiac output syndrome and pose a high risk of death [11, 13]. In our study, the incidence of myocardial ischemia was as low as Yang and colleagues’ study [5]. This perhaps attributed to that a substantial portion of patients with myocardial ischemia were excluded from candidates for emergency operation. Because our institute was a tertiary referral center, the mean time between onset of symptoms and surgery was as long as 21 h. Some patients with myocardial ischemia were excluded in consideration of missing the therapeutic window and low salvage rate. The patients suffering from myocardial ischemia did have a high operative mortality up to 57.1% in this study. In hindsight, more proactive ECMO support transiting from CPB might be helpful. The overall operative mortality of the cohort was 10.5% which was lower than the literatures [11, 13] but comparable to Wang and coworkers’ research [2]. This probably attributed to the selection bias that quite a few patients suffering myocardial ischemia were excluded. In our cohort, only 1 (0.4%) patient had complex lesion of LCA. There might be a survivor bias that patients with severe involvement of LCA died before operation. There was no statistical difference between the two groups regarding operative mortality and long-term survival, especially after discharge, and this finding was similar to Kreibich’s [13] study. Most death cases occurred within 30 days postoperatively in our cohort, so the main risk for these patients lies in perioperative period.

Previous classifications [3, 12, 13] were based on morphological characteristics of involved coronary artery and was not practical for guiding management. Our new classification was based on integrity of coronary intima and available for selecting a surgical treatment strategy. Kreibich’s [13] study had noticed the importance of cardioplegia perfusion, but they didn’t categorize the patients who were not suitable for antegrade cardioplegia perfusion into an independent type group. We thought intimal integrity is a key factor of successful cardioplegia perfusion and coronary artery repair. In our opinion, a coronary artery with a ruptured intima (type C) was unrepairable although some surgeon advocated repairing coronary trunk by a patch or interposition graft [3, 13, 14]. Mobilization and repair of coronary trunk was so dangerous and time-consuming that its advantages could be negligible. On the other hand, immediate CABG allowed timely myocardial protection. Kreibich et al. [13] suggested CABG based on the same considerations. We persisted in performing CABG for complex lesion for its simplicity and efficacy, as Wang and colleagues’ suggestion [2]. In this study, the early results of the patients with complex lesion were acceptable and better than Neri’s [3] results which had a mortality of 40%.

For simple lesion, the optimal management was also unclear. Kawahito [4] recommended using CABG in all patients whereas more researchers [2, 3, 5, 13, 14] suggested local repair. It was not easy to compare the results between different management groups for lack of a proper endpoint. So we only described the type and management of each coronary artery. In our cohort, 157 coronary arteries with simple lesion didn’t receive any management, this was quite different from previous studies [13–15] in which all the involved coronary arteries were repaired. In our cohort, eleven unrepaired coronary arteries were involved again in the same operation. Essentially speaking, recurrent coronary artery involvement is most likely caused by re-emerging dissection at aortic root. Unrepaired coronary orifice was easy to be compressed by high-pressure blood in false lumen and led to ischemia. Although the incidence of re-involvement was not very high, a potential risk did exist. We suggested that all the simple lesion should be repaired. When aortic root needs to be replaced due to its enlarged diameter, coronary ostium could be reimplanted as exact repair. In Kreibich’s study [13], they had a high rate of aortic root replacement of nearly 40% and it was about 27% in our study. We thought the decision-making of aortic root replacement should not swayed by coronary artery involvement. In fact, when coronary ostium was dissected circumferentially with a normal aortic root diameter, single sinus could be replaced with a patch and coronary ostium could also be repaired via reimplantation. In our cohort, we successfully performed right sinus replacement and ostial reimplantation for 4 patients without adverse events.

For the patients accepted CABG for simple lesion, there was no heart-related death or event. During follow-up, there was a low reintervention rate due to heart and PCI was used for only 3 patients because of newly emerging CAD. No patients need reintervention on vein graft or involved coronary artery. From our data, it seems that CABG did no harm. But it was interesting that vein graft onto arteries without atherosclerosis still had a high occlusion rate, especially when the ostium was not oversewn. A competitive flow from native coronay artery might promote this process because the vein graft anastomosed to prosthetic vessel was too long. So it is still important to reserve antegrade coronary blood flow. CABG might be useful when the tissue was not solid or the surgeon is not confident. Of course, because of its simplicity and efficacy, CABG could be a remedy approach when coronary artery was involved again, as Imoto et al. [11]and Kreibich et al. [13] recommended.

In summarization, our classification and surgical protocol of coronary artery involvement were listed in Table 4.

**Table 4:** Classification and surgical protocol of coronary artery involvement

Classification	Description	Cardioplegia	Repair strategy	CABG recommendation
Simple				
S1	Dissection around ostium with intact intima around ostium	Direct antegrade perfusion via ostium	Ostial repair or ostial reimplantation when root/sinus is replaced	CABG might be a viable alternative
S2	Dissection extending to coronary trunk with intact intima			
Complex				
C1	Dissection extending to coronary trunk with distal reentries	Retrograde or SVG perfusion	Oversew the ostium and CABG	Yes
C2	Complete or partial detachment of coronary trunk			

CABG: coronary artery bypass grafting; SVG: saphenous vein graft

This study has some important limitations. Firstly, it is a retrospective study with information bias and we didn’t include enough patients with myocardial ischemia that selection bias did exist. Secondly, we failed to compare the results of different management groups for simple lesion. Third, radiographic follow-up duration was relatively short and irregular, the results could be improved by longer follow-up time. Nonetheless, we still believe that the characteristics, treatment methods and results of coronary artery involvement have been comprehensively presented, which has considerable reference significance.

## Conclusion

The new simple classification of coronary artery involvement was available for guiding surgical management. Intimal integrity is a key factor of successful cardioplegia perfusion and coronary artery repair. Complex lesion could be treated by CABG timely and effectively. Simple lesion could be treated by ostial repair or reimplantation with satisfactory results and unrepaired coronary artery was at a risk of re-involvement. Most patients had good long-term results after they experienced the operation safely. Vein graft onto arteries without atherosclerosis still had a high occlusion rate.

## Electronic supplementary material

Below is the link to the electronic supplementary material.

Additional File Table1: Detailed information of patients who received unplanned CABG.

## Data Availability

The datasets used and/or analysed during the current study are available from.
