# Supplementary material for: A novel classification, management and long-term outcomes of coronary artery involvement in acute aortic dissection
Source: BMC Cardiovasc Disord. 2023 Jun 21;23:313. doi: 10.1186/s12872-023-03301-z (PMC10286453; doi:10.1186/s12872-023-03301-z)
Supplement: Supplementary file 1 — Additional File Table1: Detailed information of patients who received unplanned CABG. [file 12872_2023_3301_MOESM1_ESM.docx]

Supplementary Table I Detailed information of patients who received unplanned CABG

| No. | CAD | Preoperative MI | Description of involved coronary artery | Management of aortic root | Management of CA | Target vessel | Flow（ml/min）/ PI | Prognosis |
| --- | --- | --- | --- | --- | --- | --- | --- | --- |
| 1 | No | No | Dissection around ostium, ≥ half circle | No | No | RCA | Not measured | Live |
| 2 | No | No | Dissection around ostium, ≥ half circle | No | No | RCA | 36/5.6 | Live |
| 3 | No | No | Dissection around ostium, ≥ half circle | Adventitial inversion | No | RCA | Not measured | Live |
| 4 | No | No | Dissection around ostium, ≥ half circle | No | No | RCA | Not measured | Live |
| 5 | No | No | Dissection around ostium, ≥ half circle | Neomedia | No | RCA | Not measured | Live |
| 6 | No | No | Dissection around ostium, < half circle | No | No | PDA | Not measured | Death |
| 7 | No | No | Dissection around ostium, < half circle | No | No | RCA | Not measured | Live |
| 8 | No | No | Dissection around ostium, < half circle | No | No | RCA | 52/2.7 | Live |
| 9 | No | No | Dissection around ostium, < half circle | Adventitial inversion | No | RCA | Not measured | Live |
| 10 | No | No | Dissection around ostium, < half circle | No | No | RCA | Not measured | Live |
| 11 | No | No | Dissection around ostium, < half circle | No | No | RCA | Not measured | Death |

CABG, coronary artery bypass grafting; CAD, coronary artery disease; MI, myocardial ischemia; CA, coronary artery; RCA, right coronary artery; PDA, posterior descending artery; PI, pulsatility index
